# Supplementary material for: Evaluating the validity and reliability of the Tswana adaptation of the MOS-HIV tool for health-related quality of life among HIV Sub-populations in Botswana: A study protocol
Source: PLoS One. 2024 Oct 11;19(10):e0311904. doi: 10.1371/journal.pone.0311904 (PMC11469511; doi:10.1371/journal.pone.0311904)
Supplement: Attachment — Submitted filename: Responses to reviewers_PLOS One_0224.docx [file pone.0311904.s001.docx]

Thank you for the comprehensive feedback provided by the reviewers. We have carefully reviewed each comment and made significant revisions to address the concerns raised. Here are the key updates made to improve the manuscript

|  | Journal requirements comments | Authors’ responses |
| --- | --- | --- |
| 1 | 1. Please ensure that your manuscript meets PLOS ONE's style requirements, including those for file naming. The PLOS ONE style templates can be found at  https://journals.plos.org/plosone/s/file?id=wjVg/PLOSOne_formatting_sample_main_body.pdf and  https://journals.plos.org/plosone/s/file?id=ba62/PLOSOne_formatting_sample_title_authors_affiliations.pdf | We have diligently formatted the body of the manuscript to adhere as closely as possible to the prescribed format provided by the PLOS One guidelines. We understand the importance of consistency and readability in scientific publications, and we have made every effort to ensure that our manuscript meets these standards |
| 2 | We note that you have provided funding information that is not currently declared in your Funding Statement. However, funding information should not appear in the Acknowledgments section or other areas of your manuscript. We will only publish funding information present in the Funding Statement section of the online submission form.  Please remove any funding-related text from the manuscript and let us know how you would like to update your Funding Statement. | Pls add the statement below to funding statement.  OO’s research protected time was partly supported by the Incentive Funding for Rated Researchers' Grant from National Research Foundation (No:132385). Research reported in this publication was partly supported by the South African Medical Research Council (SAMRC) through its Division of Research Capacity Development under the Research Capacity Development Initiative from funding received from the South African National Treasury. The content and findings reported/illustrated are the sole deduction, view and responsibility of the researcher and do not reflect the official position and sentiments of the funders. |
| 3* | Please amend either the title on the online submission form (via Edit Submission) or the title in the manuscript so that they are identical | Thank you for bringing this to our attention. We have rectified the inconsistency between the title on the online submission form and the title in the manuscript to ensure they are now identical. We appreciate your diligence in ensuring the accuracy and coherence of our submission |
|  | Reviewer’s comments | Author’s responses |
|  | Reviewer 1  Thank you for submitting your research protocol for your study to PlosONE. Unfortunately, I found this manuscript to not meet the minimum quality standard for a study protocol. I can only advise to - consider using guidelines, e.g. the COSMIN guidelines for the development and validation of patient-reported outcome measures under www.cosmin.nl - consider accessing textbooks on the subject matter, whether by the COSMIN group (see "Measurement in Medicine" by Henrica De Vet and co-authors, 2011) or even good review articles in seminal journal (i.e., the Lohr/ISPOR guidelines), - consider using guidelines by the MAPI trust for the translation of the tool to your language/culture  Just a few pointers: - Cronbach's alpha is NOT reliability. For reliability, you want to assess inter-rater or test-retest reliability or both. Both should be done using ICCs or kappa coefficients. - Cronbach's alpha without a hint as to the factor structure of the tool is unintelligible. Also, Cronbach's alpha is not defined for multidimensional scales (see Tenko Raykov's work). When adapting a well-known instrument, you at least want to confirm its factor structure, ideally using measurement invariance techniques. - Logistic regression analysis in a cross-sectional sample with an ill-specified sampling mechanism (what you do is not "systematic random sampling", by the way: "systematic random sampling" does not exist - please look into any textbook on sampling mechanisms) and formulating this as "predictors" is not an appropriate technique for a validation study. Please reconsider the definition and subtypes of construct validity, see the COSMIN guidelines on how this should be accomplished. - Presenting a study protocol for a validation study without describing the tool or the validation studies on the tool that have gone before is a feat in itself! I think I have never come across a validation study or protocol that has not described what the tool is actually measuring. You also would want to give a history of its validation journey and - in particular - where you see the main cultural issues when translating into sub-Saharan languages/culture. - For this part in particular, you would want to consider using cognitive interviewing techniques and most likely quite extensive qualitative techniques to make sure all items, the construct, and the measurement scales are understood the same or need adapting in your culture.  As inspiration, I can highly recommend Eve Namisango's work from Uganda - please have a look into how she accomplished translation work in palliative care . | We have considered COSMIN guidelines and it emerged that whereas COSMIN guidelines provided further elucidation on the distinction between validity and reliability and their measurements, we found a misfit in their relevance to(i) non-clinical trial settings (ii) for an already defined core outcomes set, therefore we incorporated a translation and validation process prescribed by the MAPI trust, as you advised. We also consulted other resources for guidance e.g. [https://www.ncbi.nlm.nih.gov/pmc/articles/PMC5463570/#:~:text=Saudi%20J%20Anaesth.,and%20Abdullah%20Sulieman%20Terkawi]  While we acknowledge your comment on Cronbach’s alpha, we have come across numerous statistical sources that identify Cronbach’s alpha as a measure of internal consistency reliability. Perhaps, we should have been more comprehensive an indicate that we would measure internal consistency reliability using Cronbach’s alpha.[ Here are a few sources to highlight this…..  [Vaske JJ, Beaman J, Sponarski CC. Rethinking internal consistency in Cronbach's alpha. Leisure sciences. 2017;39(2):163-73.]  Hajjar S. Statistical analysis: Internal-consistency reliability and construct validity. International Journal of Quantitative and Qualitative Research Methods. 2018;6(1):27-38.  We will use “systematic sampling” to comply with your preference however, it is to be recognized that the two terms; systematic random sampling and systematic sampling refer to the same thing and often used interchangeably. In fact, the former is the more complete nomenclature to emphasize that the sampling technique belongs to the probabilistic or random class as opposed to the non-probabilistic/ non-random class. I refer you to the article by Peregrine at Lawrence University and Santa Fe Institute who provides further elucidation on this issue[ https://www.researchgate.net/profile/Peter-Peregrine/publication/247987195_Sampling_in_archaeology/links/5d9606a3299bf1c363f5708b/Sampling-in-archaeology.pdf , manual by Alvi, Mohsin (2016): *A Manual for Selecting Sampling Techniques in Research.* Herein, we cite a few books and articles in high impact journals that have used “systematic random sampling”.  **Books**   1. **A Sample of Sampling Definitions** [**Philip Bobko**](https://www.tandfonline.com/author/Bobko%2C+Philip)**,**[**Shari Miller**](https://www.tandfonline.com/author/Miller%2C+Shari) **&** [**Richard Tusing**](https://www.tandfonline.com/author/Tusing%2C+Richard)**[Pages 157-159 \| Published online: 20 Nov 2009** 2. **(1)**   1. Fuller WA. Sampling statistics: John Wiley & Sons; 2011.  **Articles**  N Engl J Med 1993; 329:661-663/ DOI: 10.1056/NEJM199308263290914  Breast Cancer Research and Treatment 1995 Vol. 36 Issue 1 Pages 55-60/DOI: 10.1007/BF00690185  <https://doi.org/10.1007/BF00690185>  J Neurosci Methods 2009 Vol. 180 Issue 1 Pages 77-81  Accession Number: 19427532 DOI: 10.1016/j.jneumeth.2009.03.001  <https://www.ncbi.nlm.nih.gov/pubmed/19427532>  Unless missed, we stated that beyond the validation process, we will apply logistic regression model where upon the HRQoL scores people will be dichotomized into “poor” or otherwise health related quality of life based on the scores and evaluate a set of independent variables as potential predictors of poor HRQoL. To make it clear, validation process, precedes regression modelling and the two are in different phases of the study.  We have reconditioned the introduction to give a consolidated history of the tool’s validation journey, to capture the pertinent contextual nuances.  **Tool Description and Validation History:**   - We have included a thorough description of the MOS-HIV tool, its validation journey in various settings, and the cultural considerations relevant to its adaptation to the Setswana language and culture. - Additionally, we have incorporated insights from Eve Namisango's work in Uganda to inform our approach to translation and validation studies in palliative care.   **Statistical Techniques and Sampling Procedures:**   - We have addressed concerns regarding statistical techniques by ensuring that appropriate methods are employed for reliability and validity assessments, including inter-rater reliability, test-retest reliability, and confirmatory factor analysis. - Sampling procedures have been revised to clarify the rationale behind the selection methods and to ensure adequate representation of urban and rural populations. |
|  | Reviewer 2  This is a much needed study within the geographical area of Southern Africa where test translation and validation studies are not as prevalent. Much research is needed on psychological measures, as well as the neglected area of the well-being of those infected with HIV. The student and their promotor should be commended on this exciting proposed study. The document reads very well, and all the important points and considerations are covered, in my opinion. The projected sample sizes are also good. All I would suggest is that they add the full stops at the end of sentences (eg. lines 94, 161, 181, 253, 256, and 260). Apart from that I wouldn't change anything, as the protocol is specific enough, but allows adequate room for statistical exploration | **Thank you very much for your constructive comments and compliments. We have incorporated your suggestions in the revised manuscript** |
|  | Reviewer 3  The authors did not desribe whther they are going to adapt the tool or they are jsut translating the 35 item tool into Setswana and use it as it is. Overall, the authors did not describe their methodology fully for example, on assessment of face and content validity, who are the experts who will be involved. the steps for the translation is also not fully described. who are the experts to be involved. When calculating the sample size for the validation process, what was the rationale for using a Cronbach's alpha of 7.0 when the reccommended is 8.0. It will be good to itemise each validation step and then state the corresponding methods e.g. (I) translation (ii) reliability assessment (iii) validity assessment | **Thank you for your comments, and we acknowledge the suggestions, and have included more details on measurements on validity and reliability. We believe the details shared are now sufficient for comprehensives and reproducibility. We have further expounded on the translation process-the approach, whom it would involve etc**  **We plan to adapt, and therefore we will assess the translated tool for its validity and reliability.**  **Methodology Clarifications:**   - We have provided a more detailed description of the methodology, including the steps involved in the translation process, experts involved in face and content validity assessments, and the rationale behind sample size determination. - The validation steps have been itemized, and corresponding methods have been stated clearly to enhance transparency and clarity.   Thanks for suggesting this article [ https://www.ncbi.nlm.nih.gov/pmc/articles/PMC5463570/#:~:text=Saudi%20J%20Anaesth.,and%20Abdullah%20Sulieman%20Terkawi ] which we have utilized to guide us through the process of translation and validation process of an existing questionnaire or tool  Nunnally J. Psychometric Theory. New York: McGraw-Hill; 1978., referenced in the article above indicates that in practice a Cronbach’s alpha of at the least 0.7 has been considered adequate internal consistency. |

We believe that these revisions significantly enhance the quality and rigor of the manuscript, aligning it more closely with the journal's standards and expectations. We are grateful for the constructive feedback provided by the reviewers and remain committed to addressing any further concerns or suggestions for improvement.
