# Supplementary material for: Evaluating the validity and reliability of the Tswana adaptation of the MOS-HIV tool for health-related quality of life among HIV Sub-populations in Botswana: A study protocol
Source: PLoS One. 2024 Oct 11;19(10):e0311904. doi: 10.1371/journal.pone.0311904 (PMC11469511; doi:10.1371/journal.pone.0311904)
Supplement: Attachment — Submitted filename: Response to Reviewers_II.docx [file pone.0311904.s002.docx]

2^nd^ September 2024

Editorial Manager

PLOS ONE

San Francisco, California

United States

Dear Sir/Madam

RE: Response to Reviewers

Thank you for reviewing our manuscript titled “Evaluating the Validity and Reliability of the Tswana Adaptation of the MOS-HIV Tool for Health-Related Quality of Life among HIV sub-populations in Botswana: A study protocol”, PONE-D-23-31341R1. We appreciate the reviewers’ for taking their valuable time to make in-depth analyses and insightful inputs which will immensely improve the manuscript.

The Reviewers’ comments are addressed below:

| **Comment number** | **Reviewer comments** | **Response to the reviewers’comments** |
| --- | --- | --- |
| 1 | Line 89: a bracket without content, probably authors missed a citation | The omission has been observed, and a relevant citation has been inserted, citation #7 |
| 2 | Line 85-87: In addition to the citation, I will suggest that authors include the MOS-HIV tool as a supplementary file for easy referencing by readers | Thank you for raising this. The MOS-HIV tool has been appended as a supplementary file for ease of referencing. Check the supplementary section. |
| 3 | Line 130: ….’’Understanding HRQoL changes during treatment, particularly for conditions like 130 cryptococcal meningitis, is crucial for improving patient care and outcomes | The relevant citations supporting the statement have been included, citations #20,21 and 22 |
| 4 | Study Design and settings - Study location: where is Princess Marina Hospital located in Botswana? Include the name of town and county or state | Additional information has been added to better describe the location of Princess Marina Hospital relative to Botswana, lines 147-148 |
|  |  |  |

We would like to believe that with the above comments addressed, the manuscript will be acceptable.

Yours sincerely,

Dr. Mooketsi Molefi
